# Reproductive cessation and post-reproductive lifespan in Asian elephants and pre-industrial humans

**DOI:** 10.1186/s12983-014-0054-0

**Published:** 2014-08-12

**Authors:** Mirkka Lahdenperä, Khyne U Mar, Virpi Lummaa

**Affiliations:** 1 Section of Ecology, Department of Biology, University of Turku, Turku FIN-20014, Finland; 2 Department of Animal and Plant Sciences, University of Sheffield, Sheffield S10 2TN, UK

**Keywords:** Ageing, Age-specific fertility, Reproduction, Senescence, Survival

## Abstract

**Introduction:**

Short post-reproductive lifespan is widespread across species, but prolonged post-reproductive life-stages of potential adaptive significance have been reported only in few mammals with extreme longevity. Long post-reproductive lifespan contradicts classical evolutionary predictions of simultaneous senescence in survival and reproduction, and raises the question of whether extreme longevity in mammals promotes such a life-history. Among terrestrial mammals, elephants share the features with great apes and humans, of having long lifespan and offspring with long dependency. However, little data exists on the frequency of post-reproductive lifespan in elephants. Here we use extensive demographic records on semi-captive Asian elephants (*n =* 1040) and genealogical data on pre-industrial women (*n =* 5336) to provide the first comparisons of age-specific reproduction, survival and post-reproductive lifespan in both of these long-lived species.

**Results:**

We found that fertility decreased after age 50 in elephants, but the pattern differed from a total loss of fertility in menopausal women with many elephants continuing to reproduce at least until the age of 65 years. The probability of entering a non-reproductive state increased steadily in elephants from the earliest age of reproduction until age 65, with the longer living elephants continuing to reproduce until older ages, in contrast to humans whose termination probability increased rapidly after age 35 and reached 1 at 56 years, but did not depend on longevity. Post-reproductive lifespan reached 11–17 years in elephants and 26–27 years in humans living until old age (depending on method), but whereas half of human adult lifespan (of those reproductive females surviving to the age of 5% fecundity) was spent as post-reproductive, only one eighth was in elephants. Consequently, although some elephants have long post-reproductive lifespans, relatively few individuals reach such a phase and the decline in fertility generally parallels declines in survivorship in contrast to humans with a decoupling of senescence in somatic and reproductive functions.

**Conclusions:**

Our results show that the reproductive and survival patterns of Asian elephants differ from other long-lived animals exhibiting menopause, such as humans, and extreme longevity alone does not promote the evolution of menopause or post-reproductive lifespan, adding weight to the unusual kin-selected benefits suggested to favour such traits in humans and killer whales.

## Introduction

Senescence is commonly observed as age declines in both fertility and survival [1]-[3]. The classical evolutionary theory predicts selection against decoupling of senescence patterns between somatic and reproductive maintenance, leading to similar ageing declines in both fitness traits [2],[4]; but see [5] for contradictory empirical evidence. The existence of menopause and post-reproductive lifespan across species has attracted considerable recent interest, first, due to medical and evolutionary attention on mid-life menopause in women and, second, due to accumulating evidence of a detectable, albeit limited, post-reproductive lifespan also in shorter-lived species such as small mammals [6], birds [7],[8], fish [9], insects [10] and nematodes [8],[11]. In contrast, studies on post-reproductive lifespan in long-lived species other than humans are rare because the difficulty in obtaining longitudinal data on these species limits the sample size available on old, potentially post-reproductive individuals. Consequently, there has been long debate over whether other long-lived species show similar kind of menopause and post-reproductive lifespan as women e.g. [6],[12].

So far, the most convincing evidence of post-reproductive lifespan in non-human long-lived species comes from marine mammals such as short-finned pilot whales (*Globicephala macrorhyncus*) and killer whales (*Orcinus orca*). These species exhibit similar life-history to humans with reproductive capacity ending around 36 and 50 years and maximum lifespan reaching over 60 and 80 years, respectively, resulting in decades-long post-reproductive phases in both species [13],[14]. Recent evidence suggests that similarly to humans [15], at least in killer whales, such a life-history may result from adaptive benefits that old post-reproductive females bring to their adult offspring [16]. The evidence for long post-reproductive lifespan (or lack of it) in terrestrial mammals is far less compelling. For example, female primates appear to retain fertility close to death [17]-[19], resulting in maximum post-reproductive lifespans of a few years [6].

Elephants are particularly interesting because similarly to whales, great apes and humans, they have well-defined social networks, large brains, offspring with long dependency (weaning at 3–5 years), and are extremely long-lived [20],[21]. Their lifespan represents the upper end recorded for terrestrial mammals along with humans, with survival commonly into 60s in the wild and maximum known age >80 yrs [22]. Some have suggested that elephants experience menopause [23] and have similar post-reproductive lifespan to women [24],[25], but due to few longitudinal datasets, this remains controversial. Little data exists on Asian elephants (*Elephas maximus*) outside zoos, where females appear to cease reproducing early (oldest breeding female in Europe was 36) [26] and have lower survival [27] compared to the wild. Such a reproductive pattern may result from captive breeding schedules that artificially accelerate reproductive senescence, when prolonged non-reproductive periods lead to increases in genital pathologies and accelerated follicular loss [28],[29]. Moreover, while some field studies on African elephants (*Loxodonta africana*) suggest that females may cease reproduction at 40–50 years [23],[24],[30], others show females capable of reproducing until 60 [22],[31],[32].

Further complications result from large discrepancies between studies in the definitions of menopause and post-reproductive lifespan. First, human menopause is defined as a complete cessation of menstrual cycles and ovulation (operational menopause) [33]. However, investigating changes in ovulation frequency and hormonal profiles in wild populations is challenging, and the functional menopause preceding operational menopause is usually defined as the age at last reproduction [33]. Second, several definitions are in use for post-reproductive lifespan. In animals for which the existence of menopause is unknown and reproductive cessation cannot be directly observed, post-reproductive lifespan is often calculated simply as the population average of the difference between the ages at last reproduction and death [17],[34],[35]. To avoid problems such as including individuals which were actually able to reproduce but just died before that, the post-reproductive lifespan can also be calculated for only those individuals surviving long enough after last reproduction (based on mean inter-birth intervals and standard deviations after last birth) [9],[17]. This method also takes better into account each individual’s own reproductive history. However, this method excludes individuals with short but legitimate post-reproductive lifespan (the lower end of the distribution) [36]. Moreover, because inter-birth intervals increase markedly with age in many animals [17],[19], these approaches may overestimate the incidence of post-reproductive lifespan when older individuals have greatly increased birth spacing. Finally, comparison of populations with these measures is problematic, because they are so correlated with overall longevity of a species [36]. Recently, Levitis and Lackey presented a new measure, post-reproductive representation, to allow easier comparisons between populations and species with differential longevities: the proportion of post-reproductive adults using life-table methods [36]. In line with this, they suggested that the main feature differentiating humans from other animals is not their ability to live beyond reproductive cessation and the subsequent post-reproductive lifespan, but the markedly greater proportion of mature individuals doing so [36],[37]. Few studies exist however to compare such methods and their suitability for estimating reproductive cessation and post-reproductive lifespan.

Here we investigate changes in survival and reproduction with age and the prevalence and length of post-reproductive lifespan in humans and in Asian elephants. For elephants, we use the world’s largest (*n* = 1040 ever reproduced females) multigenerational demographic dataset covering the life-history of several generations of logging elephants in 260 timber camps in Myanmar over a century [38]-[42]. Although these elephants are semi-captive and used in the timber industry subject to set workloads, they forage unsupervised in forests during nights and official rest periods and breed with captive and wild elephants. While basic veterinary care is available to treat injuries, survival rates reflect those of wild African elephants [27]. The elephants are not provisioned or aided in mating or calving and breeding rates are therefore unmanaged by humans. We compare female elephant reproductive and survival patterns with those of humans using as an example a comparable dataset on individual-based life-histories of historical women reproducing before the demographic transition (born <1850, *n* = 5336) to illustrate similarities/differences to humans with inevitable reproductive cessation (ie. menopause) and long subsequent post-reproductive lifespan. Specifically, we examine (a) age-specific changes in fertility, survival and birth intervals, and (b) reproductive cessation and subsequent post-reproductive survival in both species. We measure reproductive cessation by the observed age at last reproduction and by using a probabilistic model of the age-specific transition to a non-reproductive state. We determine post-reproductive lifespan by several measures that have been previously used in the literature including a recently published measure of post-reproductive representation that is particularly suited for comparisons between species [36].

## Results

### Age-specific changes in fertility, survival and birth intervals

#### Age-specific fertility and survival

**Figure 1:**
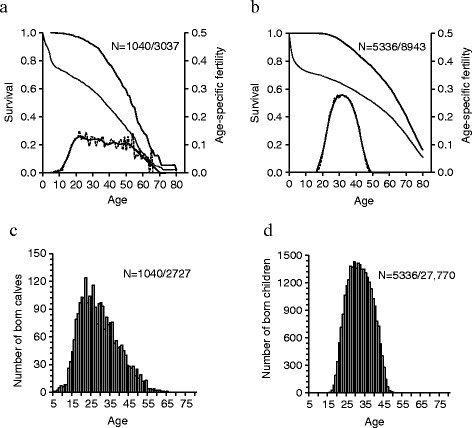
**Age-specific fertility, survival and offspring production until 80 years in Asian elephants and women. (a)** Elephant and **(b)** human age-specific fertility of ever reproducing females (dashed line) and 4-year average (solid line) shown along survival of all females born into population (elephants: *n =* 3037; humans: *n =* 8943, narrow line) and ever reproduced females only (elephants: *n =* 1040; humans: *n =* 5336, bold line). **(c)** Elephant and **(d)** human number of offspring born at each age (elephants: *n =* 2727 calves for 1040 females; humans: *n =* 27,770 offspring for 5336 women).

First, we investigated age-specific changes in fertility and overall survival in elephant and human females. Reproductive Asian elephant females in Myanmar produced on average 2.6 ± 1.76 (SD) calves (range 1–11) during their lifetime (Table 1). In general, the age-specific female fertility showed a characteristic inverted U-shaped curve but compared to humans, elephant females had lower age-specific fertility as over 20% of women reproduced at the age of peak fertility, in contrast to only >10% of elephants (Figure 1a,b). Although at the population-level the fertility of female elephants did not show a well-defined peak but instead remained at its highest at 20–50 years, most offspring born into the population were produced by females aged 20–25 years (Figure 1c) and in humans a few years later (Figure 1d). The age-specific fertility decreased after age 50 also in elephants (Figure 1a), but the pattern did not show a similar abrupt total loss of fertility in old age as in women (Figure 1b). Consequently, the species differed significantly in their age-specific probability of reproducing after age 40, with elephants experiencing a less steep loss of fertility with old age (*n =* 4989 individuals, age*species –interaction: *F*_*1,97526*_ = 144.84, P < 0.0001, *β =* 4.08 ± 0.034; age^2^* species –interaction: *F*_*1,97526*_ = 179.38, P < 0.0001, *β = −*0.051 ± 0.0038 (S.E)).

**Table 1:** Descriptive statistics of Asian elephants and humans (ever reproduced females)

	**n**	**Birth year**	**Mean afr ± S.D**	**Mean alr ± S.D**	**Mean fec ± S.D**	**Mean lifespan ± S.D**	**pr-lifespan ± S.D**
Elephant females, all	1040	1900-1993	19.85 ± 5.68 (5.30, 46.36)	32.49 ± 10.61 (7.22, 64.9)	2.62 ± 1.76 (1.0, 11.0)	38.38 ± 11.58 (8.07, 79.64)	5.89 ± 6.97 (0.00, 38.74)
Dead	320	1900-1979	19.05 ± 7.05 (5.30, 46.36)	32.52 ± 10.69 (7.22, 59.83)	2.30 ± 1.53 (1.0, 10.0)	40.40 ± 12.89 (8.07, 79.64)	7.88 ± 7.06 (0.00, 38.74)
Alive	720	1920-1993	20.10 ± 5.16 (8.00, 46.34)	32.47 ± 10.58 (8.38, 64.92)	2.77 ± 1.84 (1.0, 11.0)	37.48 ± 10.83 (10.39, 79.09)	5.00 ± 6.75 (0.00, 33.19)
Lifespan > =40	457	1911-1970	20.78 ± 6.86 (8.28, 46.36)	40.35 ± 9.16 (13.07, 64.92)	3.26 ± 2.02 (1.0, 11.0)	48.83 ± 6.87 (40.03, 79.64)	8.48 ± 8.33 (0.00, 38.74)
Lifespan > =40 + dead	154	1911-1967	21.66 ± 9.36 (8.28, 46.36)	39.77 ± 8.52 (18.92, 59.83)	2.66 ± 1.73 (1.0, 10.0)	51.09 ± 7.86 (40.03, 79.64)	11.31 ± 7.84 (0.00, 38.74)
Captive born	471	1936-1993	19.85 ± 5.68 (5.30, 46.36)	29.88 ± 9.55 (7.22, 54.00)	2.95 ± 1.93 (1.0, 10.0)	35.02 ± 10.54 (8.07, 65.11)	5.14 ± 6.68 (0.00, 34.31)
Wild born	569	1900-1986	n/a	34.65 ± 10.96 (8.38, 64.92)	2.36 ± 1.56 (1.0, 11.0)	41.16 ± 11.67 (9.46, 79.64)	6.51 ± 7.15 (0.00, 38.74)
Human females, all	5336	1595-1849	26.22 ± 5.17 (15.00, 47.00)	37.66 ± 6.27 (16.00, 52.30)	5.20 ± 3.04 (1.0, 18.0)	60.07 ± 16.50 (17.86, 100.31)	22.42 ± 15.19 (0.00, 66.18)
Dead	4529	1665-1849	26.18 ± 5.17 (15.00, 47.00)	37.35 ± 6.30 (16.00, 52.30)	5.11 ± 3.02 (1.0, 18.0)	59.78 ± 17.28 (17.86, 100.31)	22.43 ± 15.65 (0.00, 66.18)
Censored	807	1595-1849	26.45 ± 5.17 (16.00, 46.20)	39.37 ± 5.81 (17.00, 50.00)	5.76 ± 3.14 (1.0, 17.0)	61.71 ± 11.07 (20.00, 94.00)	22.35 ± 12.28 (0.00, 66.01)
Lifespan > =42	4427	1595-1849	26.50 ± 5.32 (15.00, 47.00)	38.98 ± 5.53 (17.00, 52.30)	5.53 ± 3.05 (1.0, 18.0)	65.54 ± 12.08 (42.00, 100.31)	26.56 ± 13.24 (0.00, 66.18)

Afr = age at first reproduction. Alr = age at last reproduction. Fec = number of births. Values of afr, alr, fec, lifespan (age at death or censoring) and pr-lifespan represent means with standard deviations (S.D.) and in brackets minimum and maximum values. Afr can only be calculated for captive-born elephants (sample size 38–471). Dead = individuals with known death date. Alive = individuals still alive (in elephants).

The differences in the overall survival and lifespan between elephants and humans are also clear, although the maximum lifespan reaches almost 80 years in both species (Figure 1a,b, Table 1). The age-specific survival probability of reproductive females differed statistically between elephants and humans, with survival decreasing more rapidly after age 40 in elephants than in humans (*n =* 4989 individuals, age*species –interaction: *F*_*1,120000*_ = 218.11, P < 0.0001, *β =* 0.085 ± 0.0057). For example, of all ever reproducing female elephants born into the population, 59% survived to age 50 in contrast to 75% in women. At older ages the difference becomes even more obvious as 9% of all reproductive female elephants survived to age 70, in contrast to 41% of all reproductive women (Figure 1a,b).

#### Inter-birth intervals

**Figure 2:**
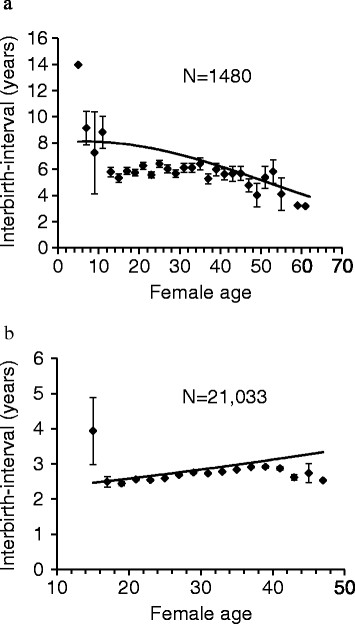
**Length of inter-birth interval with female age in elephants and women. (a)** Inter-birth intervals decreased with female age in Asian elephants (*n =* 1480), **(b)** but increased in women (*n =* 21,033) (Additional file 1: Tables S2 and S3). The figure also shows two year averages and standard errors of inter-birth intervals in raw data.

We then investigated how the age-specific changes in fertility at the population level reflected on individual-level variation in birth-intervals with age. Elephant females (*n =* 1480 intervals) produced calves on average every 5.99 ± 2.99 years (range 1.52-17.29). In elephants the inter-birth intervals decreased at older ages (age: *F*_*1,842*_ = 0.19, P = 0.66, *β =* 0.0032 ± 0.0073; age^2^: *F*_*1,842*_ = 4.15, *P =* 0.042, *β = −*0.00024 ± 0.00012; Figure 2a; Additional file 1: Table S2), in contrast to many animals such as primates in which inter-birth intervals increase with maternal age [17],[19]. The shortest inter-birth intervals were at old maternal ages (Figure 2a). The average inter-birth interval was 10.1 years for females younger than 10 years at the (first) reproduction, 6.0 years at ages 20–30 years, and only on average 4.7 years for females older than 40 years. The model controlled for significantly longer inter-birth intervals in wild-born as compared to captive-born mothers (6.20 ± 0.25 vs. 5.44 ± 0.23 years), after firstborn compared to later-born calves (5.98 ± 0.24 vs. 5.64 ± 0.23 years), for females with later age at last reproduction, as well as differences between birth cohorts and living areas (Additional file 1: Table S2). Inter-birth intervals did not differ after male and female births, with differing maternal lifespan and for censored or non-censored mothers (Additional file 1: Table S2). Age-related trends in birth-intervals might be confounded by higher infant mortality among youngest and oldest females, leading potentially to quicker replacement births among such age-groups as compared to mothers with calves to care for. We therefore also re-ran the above analysis controlling for the survival of previous calf to age 1 (*n =* 1222). The birth interval was on average 0.7 years shorter when the previous calf died early (survived: 6.00 ± 0.20 vs. died: 5.33 ± 0.31; *F*_*1,631*_ = 5.56, P = 0.019, *β = −*0.12 ± 0.050) but maternal age had a similar effect on inter-birth intervals irrespective of whether the previous calf died or survived (age*calf survival to age 1 –interaction: *F*_*1,630*_ = 0.16, P = 0.69, *β = −*0.0023 ± 0.0057; age^2^*calf survival to age 1 –interaction: *F*_*1,629*_ = 1.30, P = 0.25, *β = −*0.00055 ± 0.00048).

In contrast to elephants, the average inter-birth interval during lifetime was 2.72 ± 1.23 years (range 0.75-9.02) for women and the intervals increased linearly with maternal age (*n =* 21,033, age: *F*_*1,16592*_ = 262.59, P < 0.0001, *β =* 0.0095 ± 0.00059; Figure 2b, Additional file 1: Table S3). Such effects were controlled for significant variation in birth-interval length between parishes, birth cohorts, socio-economic classes, with poorest women having the longest intervals (rich: 2.65 ± 0.023; average: 2.79 ± 0.028, poor: 3.02 ± 0.039), birthorder, with shorter birth-intervals after firstborn than later-born (first: 2.73 ± 0.027, later: 2.91 ± 0.024), longer-living women having longer birth intervals and women with later age at last reproduction having shorter birth intervals (Additional file 1: Table S3). Birth-intervals did not differ after girl and boy births and according to whether the mother was censored or not (Additional file 1: Table S3). To control for potential effects of age-variation in replacement births, we again repeated the analysis fitting child survival to age 1 in the model (*n =* 20,656). Women whose child died under 1 had 1 year shorter following birth interval than women whose child survived beyond age 1 (1.97 ± 0.020 vs. 2.98 ± 0.024; *F*_*1,16223*_ = 3293.91, P < 0.0001, *β = −*0.42 ± 0.0073). However, maternal age had a similar effect on birth intervals irrespective of whether the previous child died or not (age*child survival to age 1 –interaction: *F*_*1,16222*_ = 0.32, P = 0.57, *β = −*0.00067 ± 0.0012).

### Reproductive cessation and subsequent post-reproductive survival

#### Age at last reproduction

**Figure 3:**
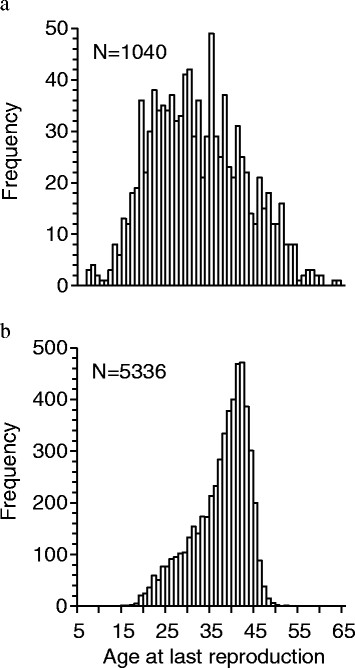
**Distribution of age at last reproduction. (a)** Asian elephants (*n =* 1040) and **(b)** women (*n =* 5336).

Next, we investigated the patterns of reproductive cessation in elephants and humans. In captive-born female elephants (*n =* 471), with known reproductive history since birth, the mean age at first birth was 19.9 ± 5.7 years (range 5.3-46.4) and the mean age at last reproduction for all females (*n =* 1040) was 32.5 ± 10.6 years (range 7.2-64.9) (Table 1). Historical women (*n =* 5336) had their first birth on average at 26.2 ± 5.2 years (range 15.0-47.0) and last birth at 37.7 ± 6.3 (range 16.0-52.3). The age at last reproduction differed statistically between the species: elephants which were still alive at the end of the study (and thus more likely to have reached old age) had significantly later age at last reproduction than humans, whereas those elephant females that had already died (many so prematurely) had on average lower age at last birth (species*censoring interaction: *F*_1_ = 34.95, P < 0.0001; species: *F*_1_ = 0.56, P = 0.45; other significant variables: censoring: *F*_1_ = 120.59, P < 0.0001; length of lifespan: *F*_1_ = 1201.80, P < 0.0001; censoring*lifespan: *F*_1_ = 165.77, P < 0.0001). This implies that survival to old age was a restricting factor for reproduction in elephants, whereas the age at last reproduction was similar in censored and non-censored humans. The variance in the age at last reproduction was significantly greater in elephants as compared to humans (Levene’s test: *F*_1_ = 226.60, P < 0.0001, β = 1.95 ± 0.13). This is illustrated with 75% of elephant females in this sample having had last reproduced before 40.0 years and 99% before 57.4 (Figure 3a) whereas 75% of women gave birth to the last child before 42.2 and 99% before 47.0 years. The maximal age at last birth in elephants was 65 years compared to 52 years in humans. Hence, the main difference in the reproductive pattern between the species concerns a more rapid loss of fertility with age in women, characterized by the last quartile in women dividing into a much narrower scale than in elephant females (Figure 3a,b), and the total cessation of reproduction occurring about 10 years earlier in women than in elephants illustrated by both the 99% quartile and the maximal age at last reproduction.

To confirm that the length of lifespan was restricting the potential to reproduce at later ages more in elephants than in humans, we also investigated reproductive cessation among only those elephants which survived beyond age 40 (calculated as the age by which 75% of females had given birth to their last offspring), and hence had an opportunity to breed at old age. These females had their last calf on average at 40.4 ± 9.2 years (range 13.1-64.9), and the age at last reproduction therefore increased by 7.9 years from the population average (Table 1). In humans, women who lived into old age (42 years; calculated as the age by which 75% of females had given birth to their last offspring) had their last child at 39.0 ± 5.5 years (range 17.0-52.3), which differs only by 1.3 years from the population average. This implies that survival rates to old age affected the reproductive pattern in elephants but not so in humans.

#### Transition to a non-reproductive state with age

**Figure 4:**
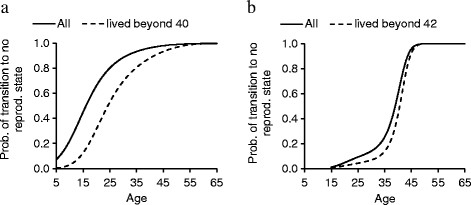
**Age-specific probability of entering a non-reproductive state. (a)** Asian elephants (*n =* 16,369 observations, 1019 females) and **(b)** women (*n =* 157,039 observations, 5176 women).

We next investigated how age affected the probability of ceasing reproduction in both species. In elephants, the cubic term of age had a significant effect on the probability of ceasing reproduction and thus the probability of entering a non-reproductive state increased steadily from the earliest age at reproduction, 5 years, reaching 0.95 at age 38 and finally exceeding 0.99 at age 49 (Figure 4a, Additional file 1: Table S4). However, the probability did not reach 1 until at 65 years. When we restricted the sample to those females which had the possibility to continue reproducing at old age (living beyond age 40, see methods for definition), the probability of entering a non-reproductive state exceeded 0.99 almost ten years later, at age 56 (Figure 4a, *n =* 457 females*;* 10,559 observations), and again did not reach 1 at any age (age^3^: *F*_*1,10538*_ = 26.81, P < 0.0001, *β =* 0.00011 ± 0.000022). In human females, the cubic term of age had also a significant effect on the probability of entering a non-reproductive state but the probability increased much more abruptly compared to elephants, from low values at age 35 (0.25), reaching 0.95 at age 45, 0.99 at age 47 and finally 1 at age 56 (Figure 4b, Additional file 1: Table S5). When we restricted the sample to those females living until old age (42 years) and having the potential to reproduce at old age, the probability of entering the non-reproductive state exceeded 0.99 at age 48 and 1 at age 56 (age^3^: *F*_*1,149000*_ = 257.22, P < 0.0001, *β =* 0.00062 ± 0.000039), at the same ages as in the entire population (Figure 4b, *n =* 4339 females*;* 149,079 observations).

#### Post-reproductive lifespan

We then investigated how long females of each species survived beyond their last reproductive event. In the whole population of Asian elephants, the average length of female post-reproductive lifespan was 5.9 ± 7.0 years (range 0–38.7; Table 1). The mean length of post-reproductive lifespan for deceased females surviving beyond 40 (calculated as the age by which 75% of females had given birth to their last offspring) years was 11.3 ± 7.8 years (range 0.0-38.7). In comparison, the length of post-reproductive lifespan among all pre-industrial women was 22.4 ± 15.2 years (range 0.0-66.2; Table 1) and in women surviving beyond 42 years (calculated as the age by which 75% of females had given birth to their last offspring) 26.6 ± 13.2 years (range 0.0-66.2; Table 1). The difference between the species in post-reproductive lifespan is illustrated in Figure 1, with the gap between fertility and survival curves of ever reproducing females at old age much smaller in elephants than in women (Figure 1a,b).

#### Post-reproductive representation

Finally, in elephants (*n =* 1040), 12.8% (PrR = 0.128) of ever reproducing adult females in the population at any time point were post-reproductive and the post-reproductive phase covered one eighth of the adult lifespan. In women (*n =* 5336), 51.2% of adult females were post-reproductive (PrR = 0.512) and hence, the post-reproductive lifespan lasted half of female adult lifespan.

## Discussion

The ability for post-reproductive lifespan appears widespread in mammals [6], although only a small proportion of all females may exhibit such a trait [37]. Elephant lifespan represents the upper end recorded for terrestrial mammals along with humans, yet reliable information on their post-reproductive longevity or reproductive termination is lacking. We used several published methods to describe the end of fertility and the prevalence of post-reproductive lifespan in Asian elephants (e.g. Additional file 1, [17],[36]) compared to humans. As in women, elephant fertility declined with age, but their pattern of reproductive cessation differed from women who terminated reproduction more abruptly and ~10 years earlier in relation to overall lifespan and had a markedly longer post-reproductive lifespan (Table 1, Figure 1). The percentage of adult females experiencing post-reproductive lifespan was 4-fold in humans as compared to elephants. These results add to the current controversy surrounding the prevalence and evolutionary origins of reproductive cessation and post-reproductive lifespan in species with different life-history and overall longevity.

We faced three difficulties whilst estimating the reproductive cessation and post-reproductive longevity in Asian elephants. First, as for most species, lack of direct data on ovulation frequency and hormonal profiles meant that we relied on age at last reproduction as a measure of functional menopause [33]. Second, because the majority of elephants were alive at the end of the study, the results may under-estimate reproductive and survival ability. Nevertheless, these estimates are to date the best available for any Asian elephant population and indeed avoid problems affecting most field studies on long-lived species where ages of all old cohorts are estimates rather than based on dates of birth. The exceptional record-keeping of the Myanma Timber Enterprise over a century provides accurate age data for all captive-born individuals, and our longevity data are thus likely more accurate than those published for wild elephants, where ages for most elephants are estimates. Moreover, if anything our finding that elephant females lack clear-cut menopause and generally maintain higher reproductive ability at old age than women is conservative, since the truncation of some elephant life-histories would produce the opposite result.

A third problem arises from the range of definitions available for post-reproductive lifespan, which all entail different problems and complicate generalisations. We tested several of such measures but overall, they all point to a lack of clear-cut menopause and wide-spread extended post-reproductive lifespan in Asian elephants. We also constructed a probabilistic model in both species to illustrate the age at which the probability of entering a non-reproductive state reached 1. In elephants, the probability increased steadily with age but did not reach 1 before age 65 years. Importantly, the probability increased more slowly in those elephants that survived to old age, which is in line with some individuals continuing to reproduce beyond 60 years. In contrast, in humans, the probability increased fast after age 35, reaching 1 at age 56 irrespective of the longevity of women. Such a difference between the species results from the restrictions that menopause poses on human women.

Using a recent alternative method for estimating post-reproductive survival [36], we found that 13% of ever reproducing adult females in the population at any time point were post-reproductive in elephants and 51% in humans. The post-reproductive lifespan covered only one eighth of adult lifespan in elephants in contrast to half of that in women. This method seems most appropriate for our study because it takes into account survival patterns of each species, is more comparable between populations and indicates the proportion of post-reproductive females in each population. Life expectancy at ages when 95% of fertility is realised gives a comparable value for post-reproductive lifespan, with 16.7 years after age 47 in elephants and 27.4 years after age 42 in humans. By any of the above method used, the length of post-reproductive lifespan in females living until old age (40/42, see methods) falls in elephants within a range of 11–17 years and in humans between 26–27 years. Consequently, the value for post-reproductive survival in elephants is greater than 10 years but less than 13% of females experience it, whereas the post-reproductive lifespan exceeds 25 years in humans and half of adult women experience it.

**Figure 5:**
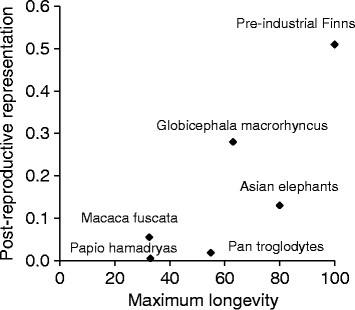
**Post-reproductive representation values plotted against maximum longevities in Asian elephants and humans compared to other long-lived mammals.** Post-reproductive representation values and maximum longevities in Asian elephants and humans from this study, other PrR-values according to Levitis and Lackey 2011 [36]. Maximum longevities in *Macaca fuscata* from [44], *Pan troglodytes* from [45], *Papio hamadryas* from [46] and *Globicephala macrorhyncus* from [47].

The PrR value of 0.13 calculated for Myanmar elephants is higher than those found in primates living in wild or semi-wild conditions (*Pan troglodytes* 0.018; *Papio hamadryas* 0.084; *Macaca fuscata* 0.055) (Figure 5) and corresponds to values found in many zoo populations of primates [36]. The value, 0.51, calculated for Finnish women correspond with patterns found in other historical or hunter-gatherer populations [36], where PrR ranges from 0.3 to 0.47 (in modern populations <0.76). The PrR-value for short-finned pilot whales (*Globicephala macrorhyncus*), known to also exhibit menopause, is 0.28 [36], which is over twice of that in Asian elephants, despite having shorter maximum longevity (Figure 5). The post-reproductive period of 14 years in short-finned pilot whales [43] is therefore potentially under stronger selection than the ~10 years in Asian elephants, because many more whales experience it compared to elephants.

Our understanding of elephant reproductive physiology and its changes with age has so far been largely based on zoo populations, which experience an absence of natural hormonal cycling [48] and high frequencies of obesity and infertility attributed to reproductive track abnormalities, post-parturient problems and accelerated follicular loss arising from repetitive and continuous non-fertile oestrous cycles [28],[29],[48]. Such zoo populations also exhibit higher mortality rates (Asian and African zoo elephants: median lifespan 19 and 17 years, respectively) than those reported for either wild African elephants (median lifespan 56 years) or for the Myanmar working Asian elephants (median lifespan 42 years) [27]. The Myanmar timber elephant dataset provides detailed life-history data of known individuals that live in their natural habitat and breed with wild conspecifics. Nevertheless, workload may reduce their fertility as compared to wild counterparts. Importantly, however, workload may only reduce reproductive rate of prime-aged but not old females, given females enter workforce at 17 years and all females >54 years are retired from work, but their logbooks are maintained until death. Therefore, the potentially lower reproductive rate of the timber elephants should not prevent us from observing continued reproduction at old age (or menopause, should this exist).

One of the few other studies available on Asian elephants on a captive Indian population reported that females aged 50–55 years reproduced as frequently as the younger cows, although a decline occurred beyond this age [20]. Also, the African elephants in Amboseli showed a drop after 50 years despite that the majority of females estimated to be over 50 years have been seen in oestrus and several reproduced close to death, the eldest mother being potentially over 60 years [21],[22]. The percentage of reproductively active females (pregnant and⁄or lactating) declined after age 50 also in Kruger National Park of South Africa, but females were capable of producing calves until close to an estimated age of 60 years [31],[32]. These findings seem to mirror our results of fertility patterns in Asian elephants. We did not find any previously published estimates of post-reproductive period for either African or Asian elephants other than claims that female elephants can have a significant post-reproductive lifespan [6],[49], and our study may thus provide the first quantitative data on this.

Overall, no comparable data exists to date on the fertility and survival patterns of very long-lived mammals with a similar lifespan to humans, except for two species of whales, which are known to have abrupt termination of reproduction (menopause) in relation to their long lifespan. Elephants seem to be clearly different from these species. For example, in killer whales, the fertility pattern with age resembles human pattern, peaking around age 20 and dropping sharply close to zero around age 50 [14]. Also the survival of killer whales at old age is remarkably similar to humans as >20% of females survive beyond 70 years, in line with 27% in humans (of all females born into the population). That inter-birth intervals decreased with female age in Asian elephants, whereas they increase in humans and killer whales [50] as well as in short-finned pilot whales [43] suggests that the elephant reproductive senescence patterns are ultimately different from those of the species exhibiting menopause.

## Conclusions

We conclude that whatever the method for estimating the duration and prevalence of post-reproductive survival, many older elephants are capable of reproducing despite fertility generally decreasing with age. The complete, irreversible cessation of reproductive capacity seen in some long-lived social animals such as pilot and killer whales [14],[43] and humans around age 50 is not perceivable in elephants. Instead, similarly to many other animals including non-human primates [51], decline in elephant fertility occurs in parallel with declines in survivorship and overall health. Our study provides the first quantitative assessment of reproductive termination and post-reproductive lifespan in elephants. The results suggest that species with same longevities do not necessarily show similar ageing patterns [8], but instead, unusual kin-selected benefits may drive the ageing patterns both in humans [15],[52] and in killer whales [16]. In these species, two types of indirect fitness benefit of reproductive cessation and post-reproductive longevity have been highlighted: adaptive benefits that old post-reproductive females bring to the success of their adult offspring [15],[16] and benefits that menopause provides for avoiding reproductive competition between generations within multi-generational families [52]. Although elephants, too, have a family system where kin help in raising offspring is pronounced and females live in multi-generational family groups [20],[21], our results show that in contrast to humans and killer whales, Asian elephant females retain the ability to reproduce until close to death. This suggests that different factors may have driven the evolution of their exceptional longevity.

## Methods

### Study populations

#### Asian elephants

The extensive demographic dataset available on semi-captive Asian elephants from Myanmar has been collated from elephant log-books and annual extraction reports archived and maintained by the Myanma Timber Enterprise. State ownership of thousands of elephants enables recording data of all registered elephants on: registration number and name; origin (wild-caught or captive-born); date and place of birth; mother’s registration number and name; method, year and place of capture (if wild-captured); dates and identities of all calves born; date and cause of death or last known date alive. The log-books are maintained and updated by local veterinarians and extraction managers at least bi-monthly to check individual elephant health and working ability. Between-individual variation in workload or rest periods is limited by law: all state-owned elephants are subject to the same regulations set by central government for hours of work/week, working days/year, and tonnage to extract/elephant. The ages of captive-born elephants are known precisely from dates of birth, whereas wild-caught elephants are aged by comparing their height and body condition at capture with captive elephants of known age, as well as many other physical features varying with age in Asian elephants such as wrinkling, depigmentation and ear folding [53]-[56]. The extent of depigmentation (freckles) on trunk, face and temporal areas, and the degree of folding of the upper edge of the ear increase with age, while hairiness of the tail tuft and degree of corrugation or wrinkliness of the skin reduce with increasing age. The Myanmar elephant catchers and trainers take careful consideration of all physical features in estimating age of wild-caught elephants. The method is considered relatively accurate, and the error in these estimates is likely to be within one year for young animals (under 20), which form the majority (68%) of those captured from the wild [57].

The entire studbook currently includes 8759 elephants born and/or captured 1900–2000; data available for this study from 2000 onwards only includes updated calving/survival status information for 207 adolescent/adult elephants and 639 calves born 2000–2012. Of all 4742 females (of which many died before reaching reproductive age), 1463 reproduced by 2000/2012. We excluded wild-caught individuals captured before 1952 because only limited records were available prior then. We also excluded 18 individuals with erroneous age at first or last reproduction, lifespan or calving date. The remaining sample includes 1040 females (471 captive-born and 569 wild-born) of which 320 had already died before 2000/2012 whilst 720 were still alive in 2000/2012 (Table 1). These females delivered 2727 calves by 2000/2012. The maximum lifespan in the sample for captive-born females is 65 years, died in 2006. The oldest wild-born female died in 1995 at 79.6 years. These elephants come from 31 timber extraction areas within ten regions in Myanmar: Ayeyarwaddy, Bago, Chin, Kachin, Magway, Mandalay, Rakhine, Sagaing, Shan and Tanin.

#### Historical Finnish people

To compare the patterns in Asian elephants with those in human populations living without access to modern medical care and contraceptive methods, we calculated age-specific survival and reproductive patterns (see Table 1) for pre-industrial Finnish women whose life-history was collected using historical population registers. Although the used human population was an agriculture rather than hunter-gathering based economy, it serves as a suitable comparison to the elephant patterns, given that the obtained values reflect those published for other traditional human populations [36] and the large sample size available for this population allowed enough old individuals being included in the study. The Lutheran Church was obliged by law to submit census registers of all births, marriages and deaths in each parish since 1749 [58]. Our data contain 5336 ever reproduced women from eight farming/fishing communities (Hiittinen, Ikaalinen, Jaakkima, Kustavi, Pulkkila, Rautu, Rymättylä, Tyrvää) with complete life-history records (*n* = 27,770 offspring). These women were born 1595–1849, and the study period therefore ended before industrialism, healthcare improvements and modern birth-control methods influenced survival and reproduction in Finland [59]. Information on husband occupation (when children were born, e.g. landowner, craftsman, tenant farmer, servant etc.) allowed categorisation of the family socio-economic status (wealthy, average or poor), a correlate of resources available [15].

### Statistical analyses

We used the following measures to investigate reproductive output and survival in Asian elephants in comparison to humans: (a) age-specific changes in fertility, survival and birth intervals, and (b) reproductive cessation and subsequent post-reproductive survival. Reproductive cessation was measured as an age when reproduction ceased (last birth), the probability of entering a non-reproductive state with age and the following methods to estimate post-reproductive survival: length of post-reproductive lifespan, proportion of females terminating reproduction ([17], see Additional file 1) and post-reproductive representation. Table 1 shows descriptive statistics of both datasets. All analyses were conducted using SAS (SAS Institute, release 9.3, 2002–2010). All biologically interesting interactions were tested, but omitted if not statistically significant and if the model fit was not improved according to Akaike Information Criterion.

### Age-specific changes in fertility, survival and birth intervals

#### Age-specific fertility and survival

We included all reproductive females for quantification of population-level age-specific fertility (*n =* 1040 for elephants and *n =* 5336 for humans) and all females born into the population (*n =* 3037 for elephants and *n =* 8943 for humans) for survival functions. We calculated age-specific fertility as the total number of offspring born during each year divided by the number of ever reproducing females alive at the end of each year. For age-specific fertility function we included females captured from the wild the first time at their capture age and thus the total sample size was adjusted to the actual number of females in a population at each age. For survival functions, we included those females still alive at the end of the study and those females disappearing before death as censored observations (right censoring) and captured females entering the population at the age of capture (left truncation), whereas captive born females entered the analysis at birth. We formally compared the differences between elephants and humans in their probability of reproducing and surviving after age 40 with time event analysis (also known as event history analysis) [60], including the species, female age and interactions with these two. Our dataset included all ages for all individuals which survived beyond age 40, from age 40 (for those who were alive) until the age of 65 years (latest age at last reproduction in elephants). For each age we coded whether the individual reproduced or not (1/0) and whether the individual was still alive or dead. The comparison of the probability of reproducing between the species included only ages when each individual was alive, and contained 97,532 observations from 4989 individuals (*n =* 457 elephants; *n =* 4532 women). The comparison of the probability of surviving between the species included 119,845 observations from 4989 individuals (*n =* 457 elephants; *n =* 4532 women).

#### Inter-birth intervals

For both species, we calculated inter-birth interval length following each birth across a female’s life, and maternal age at each birth in years (using information on year, month and date of birth). Lastborn and offspring without siblings were excluded from this analysis because their mothers did not have a following birth-interval, and stillbirths were excluded to focus on inter-birth intervals which are within the normal range. Elephant pregnancy lasts around 22 months and birth intervals <1.5 and >17.3 years (99% quartile of birth interval length) were therefore excluded as erroneous or outliers. Similarly, in humans, birth intervals <0.75 (9 months) and >9.03 (99% quartile) were excluded, resulting in sample sizes of 1480 births for 630 female elephants and 21,033 births for 4435 women. We investigated the effect of maternal age on inter-birth intervals using Glimmix procedure in SAS with gamma distribution and log-link. The elephant analysis included as fixed effects female age and age^2^, living area (10 levels), origin (wild vs. captive-born), lifespan (exact or censored), age at last reproduction, whether the female was censored or not, as well as offspring sex, birthorder (firstborn vs. later-born), and birth cohort (1960–1969, 1970–1979, 1980–1989, 1990–1999, ≥2000) to control for maternal quality [61] and finally, female identity as a random term to adjust for inclusion of several birth-intervals from same mothers. The human analysis included as fixed effects female age and age^2^, living parish (8 levels), socioeconomic status (rich, average, poor), and lifespan (exact or censored), age at last reproduction, whether the female was censored or not, as well as offspring sex, birthorder (firstborn vs. later-born), and birth cohort (<1760, 1760–1799, 1800–1829, 1830–1859, 1860–1899), and female identity as a random term. We also conducted analyses including the terms above and a categorical variable on whether the offspring survived to age 1 or not to control for potential variation in inter-birth intervals because of replacement births following early offspring death (sample sizes in these analyses: *n =* 1222 for elephants, *n =* 20,656 for humans). Final models were determined using backward elimination technique and AIC criterion for model fit to the data.

### Reproductive cessation and subsequent post-reproductive survival

#### Age at last reproduction

We used both all reproduced individuals (*n =* 1040 for elephants and *n =* 5336 for humans) and only those females survived until ‘old age’ (*n =* 457 for elephants and *n =* 4427 for humans) in both species to determine how the species-specific survival pattern modifies the age at last reproduction. We defined ‘old age’ as the age by which 75% of females had given birth to their last offspring (40 years in elephants, 42 in women). The females living until ‘old age’ are especially interesting because they can show the existence of menopause or, alternative, they can continue to reproduce at old age. We also analysed with the total sample (n = 6376, including 1040 elephants and 5336 humans) whether the species differed in their age at last reproduction with Glimmix procedure in SAS with negative binomial distribution and log-link. The analysis included as fixed effects the species (elephants vs. humans), lifespan (exact or censored), whether the female was censored or not and interactions between these variables. We tested with Levene’s test whether the variances of age at last reproduction were equal between the species.

#### Transition to a non-reproductive state with age

For both elephants and humans, we investigated at which age the probability of stopping reproducing, i.e. of entering a non-reproductive state, reached 1. We used discrete time event analysis and thus a dataset including all ages for each individual from the earliest age at first reproduction (5 years in elephants, 15 in humans) until the age of 65 years (latest age at last reproduction in elephants). Before and at the age at last reproduction, the reproductive state was coded as ‘0’ (still reproductive), and after the last reproduction, the reproductive state was coded as ‘1’ (non-reproductive). The time since previous reproduction (in years) at each age was calculated based on the exact birth dates of each offspring. For each age, survival was coded as survived versus died (or already dead during previous years) or missing (when the individual was censored at an earlier age). In the analyses, we only included the ages when the individual was coded as alive. We performed the analyses with pseudo likelihood estimation technique in GLMM (GLIMMIX procedure in SAS) with binary error structure and logit link function to determine the effect of age on the probability of entering a non-reproductive state in both species. First we conducted analyses on the entire populations and second we included only those females surviving until old age (40 in elephants/42 in humans, see the definition above) to investigate whether the results change when we include only those individuals who have the potential to reproduce at old age.

The elephant analysis was carried out on 16,369 data points between ages 5–65 (*n =* 1019 females; excluding those females who produced only one calf and died within the same year). The model included the effects of age, age^2^, age^3^, living area (10 levels), whether the individual was censored or had an exact death date, origin (captive born vs. wild caught), birth cohort (1900–1949, 1950–1959, 1960–1969, 1970–1979, 1980–1993), lifespan (censored or exact) and time since previous birth (Additional file 1: Table S4). The human analysis was carried out on 157,039 data points between ages 15–65 (*n =* 5176 females; excluding those who produced only one child and died within the same year and those having missing information on socioeconomic status). The model included age, age^2^, age^3^, living parish (8 levels), whether the individual was censored or had an exact death date, socioeconomic status (rich, average, poor), lifespan (exact or censored), birth cohort (<1786, 1786–1813, 1813–1833, 1833–1849) and time since previous birth (Additional file 1: Table S5).

#### Post-reproductive lifespan

We calculated the mean length of post-reproductive lifespan by subtracting the age at last birth from the age at death/censoring [34],[35] for all reproduced females, as well as for only those females that survived until old age (see above) and consequently with an opportunity to reproduce also at an advanced age.

#### Post-reproductive representation

Finally, we calculated post-reproductive representation (PrR) according to [36] to describe the proportion of adult years lived as post-reproductive and proportion of post-reproductive females. This value was calculated for the population of ever reproducing females in both species. The measure is independent of differences in infant and juvenile mortality between species and populations but dependent upon survival through the reproductive and post-reproductive periods. PrR is calculated based on life-table methods using functions evaluated at two ages: Age B, the beginning of reproductive lifespan or adulthood and Age M, the end of fecund lifespan. Ages B and M correspond to ages at which 5% and 95% of all births in a population have been realized independently of mortality, respectively. In elephants 5% and 95% fecundity is realized at ages 15 and 47, and in historical Finns at ages 21 and 42. The simplest population measure of survival beyond reproductive cessation is e_M_, remaining life expectancy of those individuals who have survived to age M. In reproductive elephants e_M_ = 16.9 years and in humans e_M_ = 27.4 years. We also calculated values for life-expectancy at age when 5% of fecundity has been realized; in elephants e_B_ = 39.5 and in humans e_B_ = 44.8. Finally, we calculated l_B_ and l_M_ corresponding to numbers of individuals surviving to ages when 5% and 95% of fecundity has been realised, respectively. In elephants l_B_ is 796 and in humans 5322, whereas l_M_ for elephants is 238 and for humans 4463. We used these values to quantify the proportion of post-reproductive adults according to the following formula:

(1) $$\mathrm{Pr}R\;=\;\frac{l_{M}}{l_{B}}*\frac{e_{M}}{e_{B}}$$

## Competing interests

The authors declare that they have no competing interests.

## Authors’ contributions

KUM collected the elephant data set, VL collected the human data set, ML and VL conceived and designed the paper. ML performed the analyses. ML, KUM and VL wrote the paper. All authors read and approved the final manuscript.

## Additional file

## Supplementary Material

Additional file 1: **Includes the methods, results and discussion for calculating reproductive termination [**[17]**], the results of the GLMM of the factors affecting on the length of the following inter-birth interval length in Tables S2 and S3, and the results of the discrete time event models of the factors affecting on the probability of entering a non-reproductive state with age in Tables S4 and S5 in elephants and historical humans.** Click here for file
