# Supplementary material for: Reproductive cessation and post-reproductive lifespan in Asian elephants and pre-industrial humans
Source: Front Zool. 2014 Aug 12;11:54. doi: 10.1186/s12983-014-0054-0 (PMC4144032; doi:10.1186/s12983-014-0054-0)
Supplement: Additional file 1: — Includes the methods, results and discussion for calculating reproductive termination [[17]], the results of the GLMM of the factors affecting on the length of the following inter-birth interval length in Tables S2 and S3, and the results of the discrete time event models of the factors affecting on the probability of entering a non-reproductive state with age in Tables S4 and S5 in elephants and historical humans. [file s12983-014-0054-0-S1.docx]

**Additional file 1**

**Proportion of females terminating reproduction**

To estimate the proportion of females that had terminated reproduction significantly before death (often used measure to describe reproductive cessation especially in primates), we followed [1] and included all females reproducing at least three times to determine whether each female had terminated reproduction based on how long they lived after their last birth. We subtracted the age at which a mother last reproduced from her age at death and divided this value by her own average inter-birth interval over lifetime plus two standard deviations, in order to encompass 95% of the distribution. Values >1 indicate mothers showing termination of reproduction and values <1 those capable of reproducing until death. We chose the mean plus two standard deviations as a very conservative value to minimize an apparent reproduction cessation being simply an overly long inter-birth interval [1]. Then we calculated the proportion of females showing termination under this definition for both species. Finally, we derived the mean length of post-reproductive period by subtracting each mother's age at last birth from her age at death/censoring for those females which had terminated reproduction as defined above. We calculated these values for all females in the population with at least three births, as well as for deceased females living to an ‘old age’ (see Methods).

We found that in elephant females, 16.1% of the whole population terminated reproduction (n = 409). These females with a reproductive termination produced their last calf aged 22-65 years, and the length of the subsequent post-reproductive lifespan was on average 12.8±4.6 years. Thus, these females did not only have a long birth-interval after their last reproduction but they likely experienced an actual reproductive termination. When including only deceased elephants reaching old age (40 years) (n = 63), 39.7% of females terminated reproduction after their last birth between ages 23 and 60. The length of post-reproductive lifespan in such old females with reproductive termination was approximately 14.4±5.4 years.

In women, 82.4% of all females terminated reproduction (n = 4040), with the last birth between 22 and 51 years and post-reproductive lifespan on average 26.3±11.9 years. When including only those women living to old age (42 years) (n = 3513), 92.6% terminated and the length of post-reproductive lifespan was approximately 26.8±11.6 years (with last birth varying between ages 22 and 51 years).

In elephants 39.7% surviving to age 40 terminated reproduction. Such estimates fall in the range of values reported for primates such as several lemurs, common marmoset (*Callithrix jacchus*) and gorilla (*Gorilla gorilla*) but are lower than in for example captive population of chimpanzees (60%) [1]. However, although the chimpanzee figure is clearly greater than that in elephants and some captive chimpanzees have been claimed to experience menopause around 35-40 years [2], declines in chimpanzee fertility are consistent with declines in survivorship and health in the wild [3] and elephants are thus also unlikely to have menopause according to this measure. In comparison, we found that over 90% of the pre-industrial women included in our sample and living until old age terminated reproduction and this is consistent with published data on other human populations with no access to modern medical care and contraceptive methods [1].

# References

1. Caro TM, Sellen DW, Parish A, Frank R, Brown DM, Voland E, Borgerhoff Mulder M: **Termination of reproduction in nonhuman and human female primates.** *Int J Primatol* 1995*,* **16(2):**205-220.

2. Videan EN, Fritz J, Heward C.B., & Murphy, J.: **The effects of aging on hormone and reproductive cycles in female chimpanzees (*Pan troglodytes*).** *Comp Med 2006*, **56:**291-299.

3. Thompson ME, Jones JH, Pusey AE, Brewer-Marsden S, Goodall J, Marsden D, Matsuzawa T, Nishida T, Reynolds V, Sugiyama Y, Wrangham RW: **Aging and fertility patterns in wild chimpanzees provide insights into the evolution of menopause**. *Curr Biol* 2007, **17:**2150-2156

**Table caption**

**Table S2.**

Results of the GLMM of the factors affecting on the length of the following inter-birth interval length in Asian elephants (*n=*1480 intervals for 630 female elephants). ALR, age at last reproduction.

**Table S3.**

Results of the GLMM of the factors affecting on the length of the following inter-birth interval length in historical humans (*n=* 21,033 intervals for 4435 women). ALR, age at last reproduction.

**Table S4.**

Discrete time event model of effects of age on the probability of transfer to a non-reproductive state in Asian elephants (Total *n*=16,369 observations (1019 females)). Estimates (positive reflect increasing risk) are provided for variables and 2-level factors.

**Table S5.**

Discrete time event model of effects of age on the probability of transfer to a non-reproductive state in historical humans (Total *n*=157,039 observations (5176 females)). Estimates (positive reflect increasing risk) are provided for variables and 2-level factors.

**Table S2.**

| Term | Estimate±SE | Statistic (*F*_Numdf, Dendf_) | *P* value |
| --- | --- | --- | --- |
| Age | 0.0032±0.0073 | 0.19*_1,842_* | 0.66 |
| Age^2^ | -0.00024±0.00012 | *4.15_1,842_* | 0.042 |
| Birth cohort | random variation | 7.69*_4,842_* | <0.0001 |
| Birth-order (first vs. later born) | 0.060±0.030 | 4.06*_1,842_* | 0.044 |
| Living area | random variation | 1.65*_9,842_* | 0.098 |
| Mother’s origin (C vs. W) | -0.13±0.029 | 20.64*_1,842_* | <0.0001 |
| Mother’s ALR | 0.015±0.0021 | 49.52*_1,842_* | <0.0001 |
| Constant | 1.27±0.17 |  |  |
| Mother’ s lifespan | 0.0037±0.0028 | 1.72*_1,841_* | 0.19 |
| Mother censoring (yes vs. no) | -0.0027±0.033 | 0.01*_1,842_* | 0.93 |
| Sex (male vs. female) | 0.0015±0.023 | 0.00*_1,841_* | 0.95 |

Terms retained in the final model are shown above the constant, whereas examples of those that were rejected from the final model are shown below it. Mother’s identity was fitted as random term.

**Table S3.**

| Term | Estimate±SE | Statistic (*F*_Numdf, Dendf_) | *P* value |
| --- | --- | --- | --- |
| Age | 0.0095±0.00059 | 262.59*_1,16592_* | <0.0001 |
| Birth cohort | random variation | 4.35*_4,16592_* | 0.0016 |
| Birth-order (first vs. later born) | -0.065±0.0075 | 74.11*_1,16592_* | <0.0001 |
| Parish | random variation | 17.86*_7,16592_* | <0.0001 |
| Socio-economic status | longest among poorest | 56.71*_2,16592_* | <0.0001 |
| Mother’s lifespan | 0.0013±0.00029 | 21.19*_1,16592_* | <0.0001 |
| Mother’s ALR | -0.0043±0.00096 | 20.15*_1,16592_* | <0.0001 |
| Constant | 0.88±0.051 |  |  |
| Age^2^ | -0.00004±0.000079 | *0.27_1,16591_* | 0.60 |
| Mother censoring (yes vs. no) | -0.013±0.012 | *1.15_1,16592_* | 0.28 |
| Sex (boy vs. girl) | 0.0027±0.0053 | 0.26*_1,16591_* | 0.61 |

Terms retained in the final model are shown above the constant, whereas examples of those that were rejected from the final model are shown below it. Mother’s identity was fitted as random term.

**Table S4.**

| Term | Estimate±SE | Statistic (*F*_Numdf, Dendf_) | *P* value |
| --- | --- | --- | --- |
| Age | 0.34±0.046 | 54.85*_1,16347_* | <0.0001 |
| Age^2^ | -0.0066±0.0013 | *24.16_1,16347_* | <0.0001 |
| Age^3^ | 0.000065±0.000012 | 28.25*_1,16347_* | <0.0001 |
| Time since prev. birth | 0.16±0.020 | *66.93_1,16347_* | <0.0001 |
| Time since prev. birth*Age | -0.0015±0.00050 | *8.85_1,16347_* | 0.0029 |
| Birth cohort | random variation | 8.25*_4,16347_* | <0.0001 |
| Censored (no vs. yes) | 0.64±0.045 | *195.56_1,16347_* | <0.0001 |
| Living area | random variation | 19.14*_9,16347_* | <0.0001 |
| Mother’s origin (W vs. C) | 0.18±0.044 | 15.99*_1,16347_* | <0.0001 |
| Lifespan (censored or exact) | -0.098±0.0035 | 789.35*_1,16347_* | <0.0001 |
| Constant | -4.092±0.53 |  |  |

Terms retained in the final model are shown above the constant.

**Table S5.**

| Term | Estimate±SE | Statistic (*F*_Numdf, Dendf_) | *P* value |
| --- | --- | --- | --- |
| Age | 1.57±0.13 | 151.14*_1,157000_* | <0.0001 |
| Age^2^ | -0.054±0.0037 | *212.84_1, 157000_* | <0.0001 |
| Age^3^ | 0.00066±0.000035 | 348.07*_1, 157000_* | <0.0001 |
| Time since prev. birth | 0.89±0.030 | *889.00_1, 157000_* | <0.0001 |
| Time since prev. birth*Age | -0.015±0.00078 | *374.90_1, 157000_* | <0.0001 |
| Birth cohort | random variation | 3.07*_3, 157000_* | 0.0265 |
| Censored (no vs. yes) | 0.19±0.030 | *38.93_1, 157000_* | <0.0001 |
| Parish | random variation | 288.89*_7, 157000_* | <0.0001 |
| Socio-economic status | random variation | 94.19*_2, 157000_* | <0.0001 |
| Lifespan (censored or exact) | -0.013±0.00072 | 320.20*_1, 157000_* | <0.0001 |
| Constant | -18.025±1.43 |  |  |

Terms retained in the final model are shown above the constant.
